# From Bright Bodies to *i*Choose: Using a CBPR Approach to Develop Childhood Obesity Intervention Materials for Rural Virginia

**DOI:** 10.1177/2158244019837313

**Published:** 2019-03-21

**Authors:** Fabiana A. Brito, Jamie M. Zoellner, Jennie Hill, Wen You, Ramine Alexander, Xiaolu Hou, Paul A. Estabrooks

**Affiliations:** 1 University of Nebraska Medical Center, Omaha, USA; 2 University of Virginia, Charlottesville, USA; 3 Virginia Tech, Blacksburg, USA; 4 North Carolina Agricultural & Technical State University, Greensboro, USA

**Keywords:** childhood obesity, clear communication, community-based participatory research, medically underserved area, health literacy, mixed methods, health communication, human communication, communication studies, communication, social sciences

## Abstract

This community-based participatory research (CBPR) project used a collaborative process to develop a culturally relevant workbook for parents of overweight children. We followed a mixed methods iterative process to assess clear communication using a CBPR approach. Materials were evaluated using readability tests, the Clear Communication Index (CCI), and the Suitability Assessment of Materials (SAM). In addition, we used surveys and focus groups to investigate parents’ perceptions and gather feedback from delivery staff using the workbook. While workbook materials maintained adequate grade reading levels, our iterative process and the use of CCI and SAM led to significant improvements in (a) clearly communicating the objectives of the program, (b) being culturally relevant, and (c) reaching a high satisfaction among users. These findings suggest that evaluative measures for written materials should move beyond readability and need to account for level of clarity and cultural appropriateness of messages. Furthermore, we found that that an iterative process to intervention’s material development using clear communication strategies while involving community members, parents, and research partners can lead to workbook materials that are culturally relevant to the target audience, and better communicate program objectives. Finally, this is a potentially generalizable process for improving clear communication of written health information materials.

## Introduction

Health literacy (HL) among the general public has become progressively more important for public health because many aspects of health care depend on understanding written information and verbal instruction (McCormack et al., 2010). HL includes addressing individual skill development as well as providing the delivery of actionable information that is easily understood in a manner appropriate to the audience (U.S. Department of Health Human Services and Office of Disease Prevention and Health Promotion, 2010). Many of the same populations at risk for limited HL also suffer from disparities in health outcomes (Berkman et al., 2011; Mantwill, Monestel-Umaña, & Schulz, 2015). Not surprisingly, both low HL and childhood obesity disproportionately affect rural and low-income populations (Paasche-Orlow, Parker, Gazmararian, Nielsen-Bohlman, & Rudd, 2005; Zahnd, Scaife, & Francis, 2009), with children from parents with low HL having greater obesity risk (Chari, Warsh, Ketterer, Hossain, & Sharif, 2014; Sanders, Federico, Klass, Abrams, & Dreyer, 2009). Thus, it is critical to determine the degree to which written materials clearly and effectively communicate health information when adapting evidence-based childhood obesity interventions for families in health disparate communities.

National initiatives have focused on incorporating health communication approaches to provide accessible information targeting individuals’ literacy and cultural preferences (National Institutes of Health, 2015; Plain Language Action and Information Network, 2010; U.S. Department of Health Human Services and Office of Disease Prevention and Health Promotion, 2010). The goal is to develop materials that attract and hold the readers’ attention, make them feel respected and understood, and motivate action (Centers for Medicare & Medicaid Services, 2010). Accordingly, a number of tools have been created to guide the development and evaluation of written materials for programs and interventions (Centers for Disease Control and Prevention, 2013; Centers for Medicare & Medicaid Services, 2010). However, despite being highly recommended (Brach et al., 2012; Koh et al., 2012), these tools are rarely used in the development of health promotion materials within a research setting.

The lack of use of health communication approaches may be one of the underlying reasons that HL emerges as a contributing factor of childhood obesity (Chari et al., 2014). Various family-based treatment interventions have been developed to address childhood obesity (Ash, Agaronov, Young, Aftosmes-Tobio, & Davison, 2017; Bleich et al., 2018) and, while all include written materials (White et al., 2013), there is limited evidence that those materials have been adapted and or developed using clear communication strategies. In addition, written materials used in efficacy studies with narrowly defined study populations may be less clear for audiences beyond the original study population (Brach et al., 2012), highlighting the potential low generaliz-ability of written materials used in efficacy trials.

A potential strategy to deliver actionable audience-appropriate information is to engage individuals, familiar with the cultural and linguistic patterns of the intended audience, representing a broad range of expertise, skills, and interests in the development and evaluation of health materials. In this context, effectively engaging the targeted community and research organizations in community-based participatory research (CBPR) approach may lead to improved health communication and the use of culturally appropriate materials (Israel, Eng, Schulz, & Parker, 2013; Lytle, 2009). In addition, a CBPR approach allows team members that interact with patients/participants on a regular basis to provide feedback on communication styles that may be more or less effective within the target population. Finally, obtaining feedback from members of the target population is an essential component in the process to ensure participant-level relevance of the written materials (Centers for Medicare & Medicaid Services, 2010).

This article describes the development of a culturally relevant workbook for parents of overweight children that used clear communication strategies to address key learning objectives from Bright Bodies (Savoye et al., 2005), an efficacious childhood obesity treatment program. To assess clear communication using a community-academic partnership approach, we used an iterative and systematic mixed methods process in the development and assessment of the intervention materials. We hypothesized that an iterative process that included the engagement of program participants and community staff in the development, evaluation, and revision of a program workbook would result in materials that were consistent with local culture (e.g., ways of thinking, communicating, and behaving specific to a given location and/or population) and clear communication strategies.

## Method

### Setting and Intervention Description

The Dan River Region (DRR) is a predominantly rural, health disparate and federally designated medically underserved area (Virginia Department of Health, 2008, 2012b) located in south-central Virginia and north-central North Carolina. The region currently has some of the lowest HL and highest rates of childhood obesity in the country (County Health Rankings & Roadmaps, 2015; Virginia Department of Health, 2012a). The Dan River Partnership for a Healthy Community (DRPHC) was formed as a community-academic partnership using CBPR principles with a primary mission to address obesity in the region. Under the larger DRPHC umbrella, clinical and community partners serving children in the region formed the Partnering for Obesity Planning and Sustainability (POPS) community advisory board (CAB) to develop programming specifically to treat childhood obesity (Zoellner, Hill, Brock, et al., 2017). This advisory board, collaboratively selected the Bright Bodies intervention, an evidence and family-based childhood obesity treatment program tested in metropolitan areas in Connecticut (Savoye et al., 2005), and adapted the content and structure for local delivery in the form of the *i*Choose program.

*i*Choose was developed based on the underlying principles and learning objectives of Bright Bodies, but differed in structure and duration, based on locally available resources to address childhood obesity (Zoellner, Hill, Brock, et al., 2017). *i*Choose is a 3-month family-based program that includes the following components: (a) biweekly 120-min family sessions over the 12-week program, including a nutrition lesson, exercise time, and behavioral skills training; (b) biweekly 25-min telephone support calls to set goals, resolve barriers, and reinforce content using the 5 A’s (Assess, Advise, Agree, Assist, Arrange), teach-back and teach-to-goal strategies on weeks between family sessions; (c) two 60-min exercise sessions per week; (d) workbooks for the parent and the child; and (e) biweekly child newsletters that reinforced content and provided fun activities.

### Clear Communication Strategies

The foundation of clear communication strategies to help produce “low barrier” health information material includes plain language and a reader-centered approach (Centers for Medicare & Medicaid Services, 2010). Plain language simplifies information without sacrificing the content or compromising the meaning. This approach gives special attention to graphic design and issues of cultural appropriateness, thereby making materials appealing to readers at all literacy levels. A reader-centered approach strives to understand the intended audiences by taking the reader’s perspective in identifying possible barriers within the written material. Most clear communication guidelines are derived from the social marketing framework and seek to improve communication of health messages (U.S. Department of Health Human Services, 1992). This framework proposes tailoring messages to fulfill the interests of those who would benefit from a behavior change and those who want to promote the desired behavior (Maibach, Rothschild, & Novelli, 2002). Messages are implemented as a systematic, continuous process driven by decision-based research in which feedback is used to adjust the message to ensure that all efforts are integrated and consistently support the intervention’s goals and objectives (Glanz & Rimer, 1997).

### Participatory Approach

We used a CBPR approach to engage community and research organizations to review, adapt, implement, and evaluate (Lytle, 2009) written materials used in the intervention. This participatory approach has been shown to reduce health disparities and enhance study relevance, validity, effectiveness, cultural sensitivity, and translation into practice (Choudhry et al., 2011; K. J. Coleman et al., 2005; Economos et al., 2007; Economos et al., 2013). The POPS-CAB was composed of academic researchers and community partners. The community and clinic partners are from the Pittsylvania/Danville Health District (PDHD), Children’s Healthcare Center (CHC), Danville Parks Recreation & Tourism, and Boys & Girls Club. Planning process and first-year experiences of the POPS-CAB were described elsewhere (Zoellner, Hill, You, et al., 2017). The CBPR approach also aligned with an important strategy to improve clear communication—the team approach (Centers for Medicare & Medicaid Services, 2010). The team approach included members from the community, engaged delivery staff, parents from the intended audience, and researchers.

### Development and Evaluation Process of *i*Choose Workbooks

**Figure 1.**
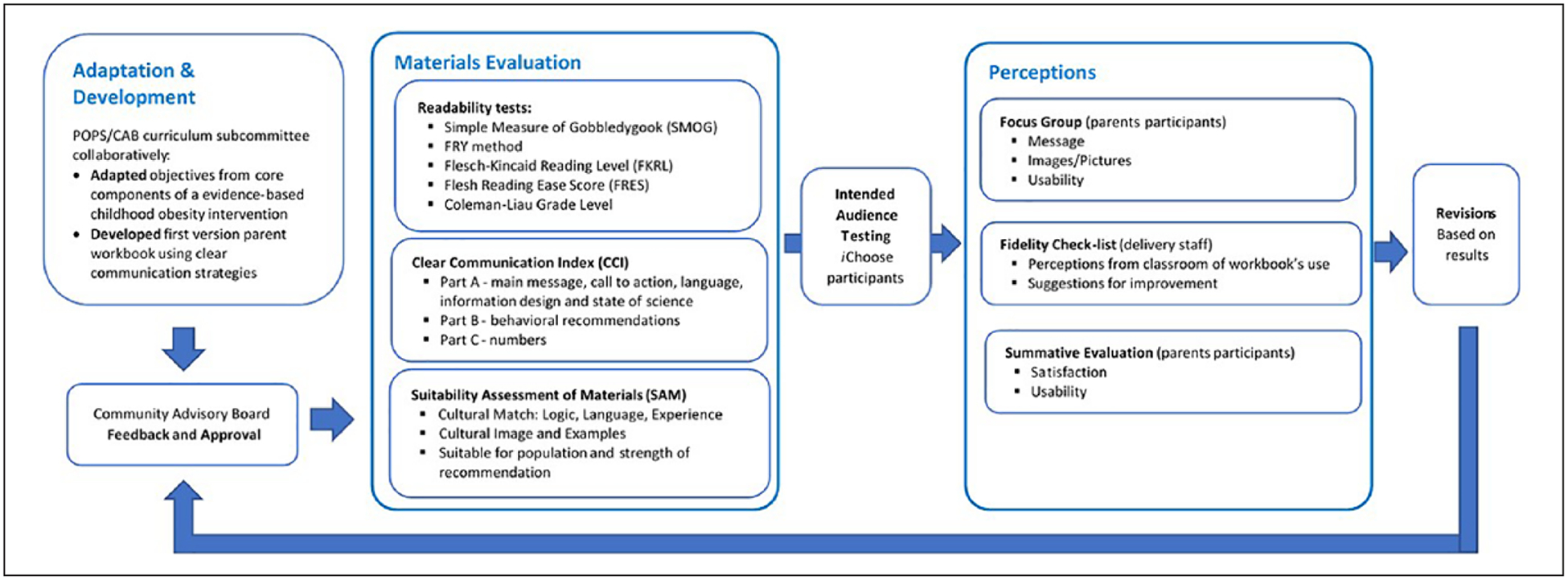
Development of workbook content using clear communication strategies. *Note.* POPS = Partnering for Obesity Planning and Sustainability; CAB = community advisory board.

One objective of the POPS-CAB was to create materials that would be relevant to local families. Thus, we designed a mixed methods approach that would engage the POPS-CAB and end users of the workbook in a process to review and adapt materials. Accordingly, we developed a formative evaluation process of the *i*Choose workbook using CBPR in a reader-centered approach. Our systematic process included a multistep process for each module to ensure that materials were appropriate for a low health literate audience (Figure 1).

The overall research design for the development and evaluation of the *i*Choose workbook was a participatory and iterative mixed method design. Due the prolonged dynamic and complex contact with the community the use of mixed methods are useful in CBPR research (Lucero et al., 2018), as it allow us to draw upon the strengths of both the depth of qualitative research and the breath of quantitative work (Fetters, Curry, & Creswell, 2013). In addition, our participatory and iterative approach allows for the use of qualitative data to provide direction for improvements in the written materials—where quantitative data indicated improvements were necessary (Fetters et al., 2013). The mixed method data collection was used to strengthen the validity of the conclusions reached by the study (Johnson & Onwuegbuzie, 2004). During the process, we performed triangulation of quantitative (Clear Communication Index [CCI], Suitability Assessment of Materials [SAM], readability tests and surveys) and qualitative (interviews, classroom feedback and focus groups [FGs]) methods from different sources (i.e., community and academic partners, delivery staff, and parents) to increase the likelihood that refined materials met the needs of participants and delivery personnel while also adhering to the evidence-based principles at the core of the program. Triangulation is considered a useful means of capturing more detail, minimizing the effects of bias, and ensuring a balanced interpretation of available data and soundness of study conclusions in qualitative studies (Jakob, 2001). Both participatory and formative evaluation approaches (Israel et al., 2013) were designed to involve the POPS-CAB members and program participants, in multiple components of the process. The intent was not only to develop an evidence-based workbook but also incorporate HL best practices to achieve a clear and effective communication with the target population. This case study occurred over a period of 3 years and is embedded within a larger CBPR pilot trial of the *i*Choose program (NIMHD-R24MD008005). The Institutional Review Board (IRB) at Virginia Tech approved this study, and all participants gave written informed consent prior to participation.

#### Adaptation and development of the workbook.

After the intervention selection process by the POPS-CAB members, community partners identified that the written materials from the selected intervention (Bright Bodies) needed adaptations to better fit their community profile, including more culturally relevant content and images as well as the need to address different levels of HL. As the Bright Bodies (Savoye et al., 2005) materials were under a copyright and could not be modified, we identified the core principles and intervention objectives from the literature and used them to develop a workbook to accompany the *i*Choose program. We, then, established a curriculum subcommittee, composed of both researchers and community partners, to develop the workbook, based on those core principles and learning objectives. Themes for the intervention modules were reviewed and incorporated as individual chapters in the workbook. Six chapters were created, one for each intervention module. Following the family class format, the chapters were divided into content areas (sections) of Nutrition, Physical Activity (PA), and Behavioral Strategies. Each chapter included the module objectives (Table 4), educational content, a class activity, and homework. The subcommittee presented a first version of the workbook to the POPS-CAB to approve and/or make suggestions and followed this with two rounds of evaluation for readability, clear communication, and cultural appropriateness (Figure 1).

#### Tools for workbook evaluation.

A *readability evaluation* of the workbook content was performed to ensure that the reading level was adequate to the proposed target population. The workbook writing aimed a fifth-grade reading level. To evaluate the reading level, we used five different measures of readability: Simple Measure of Gobbledygook (SMOG; Fitzsimmons, Michael, Hulley, & Scott, 2010), Flesch-Kincaid Reading Level (FKRL; Kincaid, Fishburne, Rogers, & Chissom, 1975), FRY method (Fry, 1968), Coleman-Liau Grade Level (M. Coleman & Liau, 1975), and Flesh Reading Ease Score (FRES; Flesch, 1948). All measures were applied in a plain document with no pictures or tables included in the calculations. We decided to use different measures for readability because each one focused on a different aspect of the text (e.g., word and/or syllable count, sentence length). While readability scores provide an estimation of grade level necessary to read the material, however, those scores do not provide information on reading ease, prominence of main messages, behavioral strategies to initiate action, or cultural relevance, and thus, they can be misleading when determining the likelihood that materials clearly and effectively deliver information. Therefore, in addition to the readability tests we performed a more comprehensive assessment of written materials that explicitly addresses the degree to which information is clearly communicated to intervention participants. After a literature review, we decided to use the CCI (Baur & Prue, 2014) and the SAM (Doak, Doak, & Root, 1994). Both evaluations were conducted by POPS-CAB team members (*n* = 14).

The CCI (Baur & Prue, 2014) was developed by the Centers for Disease Control and Prevention (CDC) to guide the development, implementation, and assessment of messages and written materials to make them easier for people to read, understand, and use. Items on the CCI aim to represent the most important characteristics to enhance clarity and aid people’s understanding of information. The CCI assesses materials in seven key areas divided into four parts: (a) Part A includes the main message and call to action, language, information design, and state of the science; (2) Part B evaluates the clarity of behavioral recommendations; (3) Part C focuses on the use of numbers and clarity of expressing numbers; while (4) Part D focuses on providing a clear description of associated risks of taking or not taking a certain action. Not all parts of the CCI are applicable to all written materials and depend on the presence or absence of information on behavioral goals, the use of numbers, or if risk factors are presented in the materials. The CCI consists of 20 items that produce a numerical score to objectively assess materials. The scores from each part were tallied to obtain an overall score (out of 100%), with a recommended standard of 90% or above to make materials easy to understand and use (Baur & Prue, 2014).

The *SAM* (Doak et al., 1994) enables reviewers to consider six categories: content, literacy demand, graphics, layout and typography, learning stimulation, and cultural appropriateness. The SAM’s score falls into one of three categories: superior, adequate, or not suitable. As the SAM is redundant, in some areas, to other assessment tools used in this study, we only used the SAM items related to cultural appropriateness, cultural images and examples, and strength of recommendation, which were not covered by the CCI.

#### Training and procedures for workbook evaluation and refinement.

POPS-CAB members (*n* = 16) completed training on the CCI and SAM evaluations. A daylong training was offered by a CDC expert and developer of the CCI instrument via videoconferencing, and a SAM presentation hosted by academic partners targeted local capacity development and shared learning between the partners. Trained POPS-CAB members (*n* = 14) were subsequently randomly assigned to conduct the evaluation individually on specific chapters of the workbook (two to three members per chapter). Six small groups composed of members of the research team (*n* = 7) and community partners (*n* = 7) that had individually assessed the respective chapters reconciled and consolidated their individual CCI ratings into a shared rating. During these small group sessions, POPS-CAB members used the materials to resolve differences in ratings. Across all groups, consensus on rating was achieved and used as the CCI value for the given chapters. As a group consensus, Parts A and B were applied to all chapters. Part C was pertinent to Chapters 1 to 3 and Part D was not pertinent to the material evaluated in this study.

#### Intended audience testing.

The workbook versions were pilot tested in the first and second wave of families enrolled in the *i*Choose program. During the first wave, the workbook was tested while it was being developed by the POPS-CAB curriculum subcommittee. At the end of the first wave, we revised the workbook and incorporated the initial feedback and results from Wave 1 creating a second version of the workbook. Parents and caregivers (Waves 1 and 2) had an average age of 40 years (*SD* = 8.5 years), were predominantly female (95%), with most having at least a high school education (91%), and nearly half having income less than US$25,000 (46%). In addition, participants completed the Newest Vital Sign (NVS) to assess HL and numeracy (Weiss et al., 2005). The NVS results in our sample indicated that approximately 34% of the parents and caregivers had low HL (Zoellner, Hill, Brock, et al., 2017).

*Summative evaluation* interviews (*n* = 38) and *FGs* (*n* = 11) were used to gather parent’s feedback on the workbook. Trained research personnel conducted both activities. The summative evaluation was completed with all parents who completed a postprogram assessment and included questions about satisfaction (e.g., “How satisfied were you with the parent workbook?) and usability (e.g., “How often did you use the workbook outside of the family class?”). For the FG, the curriculum subcommittee developed a script with CCI-based questions to guide the discussions for each workbook chapter following their objectives (e.g., “How well do you think these messages were explained in the workbook?,” “What things in the workbook helped you to better understand these messages?”). Eleven of 14 invited parents agreed to participate in the FG. To accommodate participant schedules, we conducted two small FGs and offered child care. FGs were audio-taped and transcribed verbatim to provide information on areas that contributed to potential workbook adaptations.

During the intervention period, *fidelity checklists* were also completed for each family class and included opportunities for delivery staff to provide comments about how the workbook was used during the sessions and if parents suggested adaptations. We collected all comments (open ended) related to the workbook from the fidelity checklists across two waves of intervention delivery for analysis.

#### Workbook revisions.

Following the first CCI evaluation, the curriculum subcommittee went through FG transcripts, field notes, delivery staff qualitative feedback, and CCI open ended questions, selecting quotes that indicate proposed changes. The findings were then summarized as a “proposed revision list” for each workbook chapter and chapter section (i.e., content area). The curriculum subcommittee then made adaptations to the workbook based on the revision list. The final documents were presented and reviewed by the POPS-CAB using an iterative process. Feedback from the POPS-CAB was used to confirm, correct, or clarify the changes made to the workbook.

### Analysis

The quantitative data from instruments and surveys were analyzed in SPSS version 21, and analyses included frequencies, means, standard deviations, paired *t* tests, with results presented in tabular format. Individual and reconciled ratings were summarized and “within subjects” *t* tests were used to determine differences in individual and overall CCI and SAM ratings. Though the sample size of stake-holders was small, we also compared community and academic partner ratings to determine whether differences emerged in ratings. The transcripts from the FGs and qualitative portion of the forms were reduced to meaning units by the curriculum subcommittee and inductively categorized across the areas used to evaluate and improve the workbook chapters—representative quotes were provided within the results section indicated by quotation marks and italics (Table 3). All results are presented based on the initial version of the workbook used in Wave 1 (before) and the revised workbook (after) used in Wave 2.

## Results

### Material Evaluation

#### Readability tests.

Readability tests revealed an overall workbook mean reading level to be at fifth grade. Table 1 shows results for all five tests performed in which no statistically significant changes were observed between tests conducted on the before revisions and on the after revisions. Variability between the measures of years of education required to understand the text showed results ranging from below fourth grade for SMOG (3.8) which considered the complexity of words (polysyllabic count), and seventh grade for Coleman-Liau (6.8) that considered the length of words (character count). In the Flesch Reading Score, where scores indicate on a scale of 0 to 100 how easy to read the material is, the overall result by chapters found it to be easy (80) to fairly easy (78). In addition, variability was found between chapters in ease of reading ranging from standard (62) to easy (80–86).

#### CCI.

The initial POPS-CAB CCI evaluation resulted in an overall score of 76% reflecting an inadequate clarity level (Table 2). Qualitative comments (e.g., need to address multiple main messages and include more ethnically diverse pictures) described in detail on Table 4 demonstrated the need for a revision. For the final product, the evaluation resulted in a significant improvement in overall rating with a score of 90% (*p* < .01), reaching the level where materials are considered to be clear (i.e., ≥90%). Both before and after revisions mean ratings were nearly identical between community and academic partners, as well as individual and group reconciled ratings. When considering results by workbook content area, overall ratings showed a mean increase of 19.2% (range = 5%–45%) across all chapters. Results also show improvement (μ = 13%) in all CCI component Parts. Changes to the clarity of Main Message (Part A) showed larger improvement (μ = 24%). Both before and after revisions, the workbook had strong Behavioral recommendations (Part B = 94%−100% across content area).

#### SAM.

SAM results indicate that the cultural appropriateness of material remained rated as superior (μ = 2) before and after for overall results and when considered by research members (Table 3). However, community partner ratings for the Cultural Image and Examples went from adequate (μ = 1) to superior (μ = 2). Before the evaluation, most of the comments in the SAM’s comments section were related to improving pictures to “*represent more race/ethnicities*.” This improvement can be exemplified by comments left by community members after revision “*Great improvement regarding different ethnicities/cultures*.” Strength of recommendation was strong overall for both revisions (before = 8/10; after = 9/10).

#### Results from the FGs.

FGs revealed that the workbook accomplished its objectives and was easy to understand. They also reported that the workbook helped them rethink their behaviors and influenced them to promote health changes. Furthermore, parents reported that the written materials supported the other intervention components and was used as a reference resource. Finally, FGs also revealed workbook’s areas that needed improvement in format (e.g., more visual cues and separation of sections) and content (e.g., screen time focused in all types of media not only on TV) were also highlighted (Table 4).

#### Results from the fidelity checklist.

**Figure 2.**
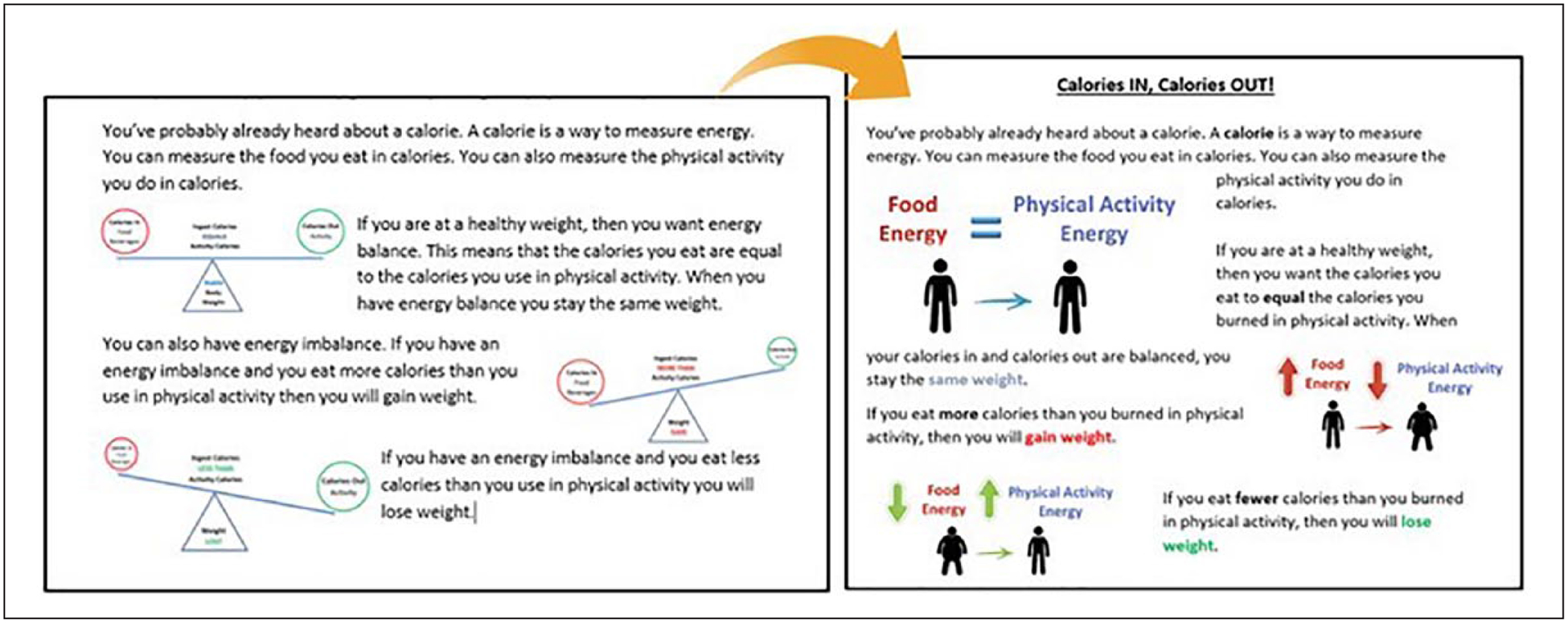
Sample of change in Chapter 1—Energy balance section based on qualitative feedback.

Delivery staff feedback revealed areas for improvement related to comprehension (e.g., difficulty in understanding energy balance) and format (e.g., Use “rounded” number to facilitate calculations). Table 4 shows sample of selected quotes by chapter from the transcripts and from the delivery staff feedback that influence changes in the workbook’s first version and Figure 2 provides a sample of changes.

#### Results from the summative evaluation.

Data gathered from the parent/caregiver summative evaluation presented no significant difference between waves. Results indicated that parents felt satisfied with the workbook (μ = 9.2/10, *SD* = 1.08) and found it to be helpful (μ = 9.3/10, *SD* = 1.5), and agreed that was easy to find information that they need in the workbook (μ = 9/10, *SD* = 1.4). Lower rates were found about the usability with parents indicating that they did not often use the workbook outside the classes (μ = 3.5/10, *SD* = 1.4), did not often use the goal setting and tracking sheets (μ = 3.4/10, *SD* = 1.5), or think they will often use the workbook after the program ended (μ = 2.8/10, *SD* = 1.6). Qualitatively, parents indicated they liked that it was easy to read and follow, used plain simple examples, and thought the illustrations were nice. Parents reported lack of time, often due to work or other commitments, as the primary barrier to using the workbooks.

## Discussion

We have described an iterative and systematic formative evaluation using a CBPR approach to develop, evaluate, and improve a childhood obesity workbook for parents of overweight children that used clear communication strategies to address key learning objectives. Because written materials are often used as an important intervention component (White et al., 2013), the main objective of this study was to offer a process guide for the development and evaluation of written materials using a collaborative approach. The study adds to the current literature by providing a process to combine available HL tools (White et al., 2013), such as the CCI evaluation system, SAM, and readability statistics using a CBPR approach.

We documented that the intervention materials developed for this study were written at a fifth-grade reading level which was below the average grade level required for our participants (>ninth grade; County Health Rankings & Roadmaps, 2015). The SAM ratings improved following revision in our study, primarily due to changes in community partner assessments. This is consistent with research on written materials targeting parents to prevent childhood obesity (White et al., 2013) where the findings from the SAM measure identified specific areas related to cultural appropriateness that reduced the overall suitability of materials in their original form. White et al. (2013) also documented superior ratings after making specific revisions in response to SAM scores. Common revisions in response to these scores included rewording passive sentences, enhancing the color schemes, reframing of health information to better coincide with typical reading patterns, and adding in culturally appropriate visuals (White et al., 2013).

This is the first study, to our knowledge, that incorporated the CCI in the evaluation of childhood obesity treatment materials for parents. The CCI added evaluative factors for written materials beyond readability statistics and cultural appropriateness, and provided actionable information to improve the original workbook materials. Consistent with Baur and Prue (Baur & Prue, 2014), revisions based on the CCI resulted in written materials that were rated higher than original materials. These changes are hypothesized to increase the likelihood of parents, regardless of their educational level, to identify and understand the main message, and interpret numbers in each workbook section. Unfortunately, this hypothesis cannot be directly tested with the current study due to the multicomponent intervention (e.g., changes in comprehension could be due to adaptations made to in-person class or telephone support sessions rather than due to workbook changes)—though this would be an excellent area for future research.

Our study findings also highlight the importance of moving beyond readability statistics as a sole indicator of the appropriateness of written materials for a given audience. It is of note that the results of the readability assessments did not change when comparing to the original and revised materials—both were ~5th-grade reading level. In contrast, both the CCI and SAM assessments provided actionable information for revisions and demonstrated significant improvements in ratings between the original and revised materials. Despite the finding that approximately 34% of the parents in our sample had limited HL (Zoellner, Hill, Brock, et al., 2017) and that 18% of the adults in the region lack basic literacy skills (National Center for Education Statistics, 2003), readability assessments would have suggested that the original materials were appropriate. However, readability scores do not provide information on reading ease, prominence of main messages, behavioral strategies to initiate action, or cultural relevance—as the CCI and SAM provide—and can be misleading when determining the likelihood that the materials clearly and effectively communicate intervention information (Centers for Medicare & Medicaid Services, 2010). Therefore, the use of clear communication strategies has the potential to enhance program efficacy, perceived cultural relevance from community members, and satisfaction among participants.

The CBPR approach that actively engaged community partners in the workbook planning and adaptation process increased community capacity related to HL. Community members of the POPS-CAB played a critical role in the design and implementation of the written materials. Incorporating CAB feedback was important to develop clear and suitable materials for the regional childhood obesity treatment program. Their involvement in the interpretation and application of the evaluation findings also enhanced the quality of the materials while developed feelings of inclusion and ownership by community partners. The engagement of community partners in training on the CCI and SAM included the added value of increasing capacity in community members and may also lead and contribute to improved organizational HL and the quality of practice (Israel, Schulz, Parker, & Becker, 2001).

An interesting finding was the similarities between community and academic reviewers where the mean ratings were nearly identical while evaluating the communication strategies (CCI). However, the differences arouse in the evaluation of the cultural appropriateness where community reviewers indicated that they wanted to see more racial and ethnic representation in the images and examples despite highly rating the adequacy of the workbook. Based on that evaluation, the curriculum committee became aware and made sure to keep this aspect in mind while addressing participants’ requests (table 4) to add more pictures and food/recipes examples to the chapters. This example highlights how the CBPR approach influenced the changes to the content reflecting the community expertise of the local context. This input improved the cultural appropriateness of the materials, which otherwise could have been unnoticed by the researchers and readability tests.

Finally, to recognize and praise the significant time commitment of our community partners in the participatory evaluation process, our approach had an ongoing emphasis on optimizing the process, for example, by adapting to the resources available and determining the minimum data necessary for workbook development. At the same time, our community partners also indicated that they valued receiving specific details about detail the process, such as detailed reports by chapter of each indicator evaluated and perceptions that led to workbook changes and adaptation.

Our study included a number of limitations. First, we did not conduct a final round of FGs to assess the final version of the parent workbook. Although the use of the FG interviews in Phase 1 contributed to understanding of the problem from a reader-centered point of view, it was extremely labor and time intensive, including time needed to conduct the analysis collaboratively with community partners. Therefore, we decided not to conduct a second round of FGs after the final revisions because the materials showed a significant improvement and reached acceptable clear communication and suitability levels. Second, the sample size of CAB members that evaluated the documents before and after revisions was small. This is due to the nature of the study and our goal to report on the process of assessment and adaptation. Third, we developed the workbook and tested it within a multicomponent intervention, which does not allow for independent comparison of changes in the workbook with comprehension and study outcomes. Still, the findings provide a process for developing clear written materials for adults from an ethnically diverse, low income, and low literate community.

## Conclusion

This article describes a CBPR approach to applying clear communication strategies in the development of childhood obesity intervention materials. The approach is driven by and tailored to community needs and involved contributions from individuals who would ultimately deliver the intervention and participants who have engaged with the intervention materials. We found that a process that included the engagement of community members and program participants in the development, evaluation, and revision of a program workbook to be both feasible to our CAB and staff and acceptable to potential participants who represented the target population. Our iterative process resulted in improved written materials that are written in an adequate grade reading level, clearly communicated the objectives of the program, and were culturally relevant while achieving a high satisfaction among users. The findings of this study suggest that, first, evaluative factors for written materials need to move beyond readability and include measures of the level of clarity of the messages and cultural appropriateness to provide actionable information to improve health information materials and that, second, an iterative process to intervention’s material development using clear communication strategies while involving community members, parents, and research partners may lead to workbook materials that are culturally relevant to the target audience, and better communicate program objectives.

## Figures and Tables

**Table 1.** Readability Tests Results Before and After Revisions.

	Measure of years of education required to understand the text								Easiness to read	
	SMOG score (grade level)		Flesh-Kincaid score (grade level)		Fry score (grade level)		Coleman-Liau score (grade level)		Flesch reading ease score (0 very confusing; 100 very easy)	
	Before	After	Before	After	Before	After	Before	After	Before	After
Overall	3.8	3.8	5.1	5.1	5.8	5.8	6.7	6.8	80	78
Chapter 1	4.1	4.3	6.1	5.8	7.0	7.0	7.3	6.9	74	77
Chapter 2	4.2	3.8	6.1	6.0	7.0	7.0	7.1	7.5	75	62
Chapter 3	3.8	3.8	4.4	4.5	5.0	5.0	6.2	6.2	85	84
Chapter 4	3.8	3.8	4.3	4.6	5.0	5.0	5.8	6.3	86	83
Chapter 5	4.1	4.0	5.0	5.1	6.0	6.0	6.9	7.0	82	81
Chapter 6	3.0	3.0	4.4	4.3	5.0	5.0	6.9	6.9	80	81

*Note:* No significant differences between the first and second version of the materials were identified. SMOG = Simple Measure of Gobbledygook.

**Table 2.** CCI “Within Subjects” *t* Test Results Before and After Workbook Revisions.

		
CCI scores	Before (%)	After (%)
Workbook CCI scores (*n* = 14)		
Overall	76**	90**
Community (*n* = 7)	77*	89*
Researchers (*n* = 7)	75*	90*
CCI scores by chapters (*n* = 14)		
Chapter 1	49	94
Chapter 2	73	88
Chapter 3	57	87
Chapter 4	69	87
Chapter 5	83	85
Chapter 6	88	93
CCI components parts scores by workbook’s content area (n = 14)						

	A—Core/main message		B—Behavioral recommendations		C—Numbers	
	Before (%)	After (%)	Before (%)	After (%)	Before (%)	After (%)
Overall	63**	87**	96**	98**	67**	80**
Nutrition	62*	88*	100	100	58	60
Physical activity	67*	86*	94*	100*	75*	92*
Behavioral	59**	88**	94	94	67**	89**

*Note:* CCI = Clear Communication Index.

* *p* < .1.

** *p* < .05.

**Table 3.** Suitability Assessment of Materials Results Before and After Revisions.

Suitability Assessment of Materials						
	1. Cultural match in logic, language and experience		2. Cultural image and examples		3. Suitable for your population?	
	Before	After	Before	After	Before	After
Overall	Superior	Superior	Superior**	Superior**	8*	9*
Community	Superior	Superior	Adequate*	Superior*	7***	9***
Researchers	Superior	Superior	Superior	Superior	9	9

*Note:* SAM = Suitability Assessment of Materials.

* *p* < .1.

** *p* < .05.

*** *p* < .01.

**Table 4.** Sample of Perceptions That Leaded Workbook Changes and Adaptation.

Chapter	Learning objectives	POPS/CAB feedback From CCI open questions	Delivering staff feedback From fidelity checklist	Parent/caregiver participant’s feedback From focus group	Workbook’s change—adaptations
Overall		More than one message Main message is not at the beginning of section. Pictures could represent more ethnicities (people and food) Some sentences are in passive voice	Include most of the intervention handouts in the workbook Change activities—Simplify to facilitate time management at classroom Change activities—Use “rounded” number to facilitate calculation	“1 think that the workbook was put together really well. It was organized and in sections, and explained each section thoroughly. It was easy to read … and we will continue to refer to it as needed.” s“Something that 1 think would be helpful is it has them in this book, but then it stays in this book … if it was on my refrigerator every time 1 opened it, 1 would be like, oh, okay.”	Reorganized content to focus in one main message with is stated in the first paragraph Changed pictures for include more diverse ethnicity. Included most of the handouts as part of the workbook. Made the tracking sheets and goal settings as “tear out” without compromising the content (blank back) Added the Tips for You section Changed remained passive voice sentences to active voice
Chapter 1	Describe energy balance and how it influences weight. Identify the food groups included in MyPlate. Identify that children should participate in 60 min of moderate to vigorous activity daily. Set a specific, time-based, and achievable goal for eating and physical activity.	[Need to] focused on energy balance	Better discussion about calories and energy balance (make clear that they need imbalance) Setting SMART goals were too intense for young kids next time have kids set 1 goal /SMART goal setting was “too much to digest” for kids	“Plain and simple.” “This explained it in real details and everything.” “[Had] anything that could be done to this section to make better” Energy balance was hard [to understand], 1 mean … We need imbalance right?”	Changed energy balance content language (energy “in-burned” to “in-out”), and pictures (changed scale to body outlines) Reduced the SMART goals to kids
Chapter 2	Choose foods to eat more often, and to cut back on foods to eat less often. Identify how to be physically active in a way that fits their preferences and lifestyle. Help their children to identify barriers and address them at daily bases without focusing overly on weight.	Pictures of sometime/anytime foods might be more helpful than words alone (there are sometimes graphics—arrows + smiley face—but no food pictures on graphics in this section. First statement could state the health foods are anytime food. May need more diverse pictures—more non- White people. [Heath snacks] Dried fruit and nuts can be expensive and people don’t know what hummus is.	Families were not using the tracking system.	“1 think it could have been a little bit more with healthy foods and choices.” “Maybe put the name of the food because that was kind of hard to tell [by the picture]” “1 actually like this [exercise] pyramid. 1 think it would be clearer and maybe if it was a tear-out and we could put in on the refrigerator it would be great” “1 would like to see on my fridge; that we could reference every day without having to go into a notebook (…) It would really make my kids more accountable and think about that.” “1 think the activity pyramid is helpful. But it’s just don’t make it so pixelated so we can actually read it. “1 like color and what 1 see is a whole bunch of black and white (…) this seems like schoolwork a lot of times (…) 1 look this part and think: Boring!” “The information is great. 1 think it just should have been more visual pictures, and then maybe added a couple of extra pages and … it’s like all this information in one place.” “They need to highlight some of the main points or something.” “1 think its [multiple choice] a good way to get started because I’ve never really set goals like this (…) And 1 think this was a good way to kind of get the ball rolling.”	Made the tracking sheets and goal settings as “tear out” without compromising the content (blank back) Changed layout to include more pictures, less text dense and more colorful. Made a new adapted version of the excise pyramid which was more clear and included race variety images. Added legend to food pictures Added an annex section with health snacks options that are quick, easy and affordable.
Chapter 3	Define the appropriate portion sizes for parents and kids. Recognize when they are hungry and when are enough. Identify ways to cut back screen time to less than 2 hours per day. Develop strategies to change home environment cues so that they support healthy eating and physical activity.	More active voice: screen time “it is recommended” is passive consider “we recommend” and take out “can” and make it clear. Calories in food not rounded. Consider breaking up the “cut back on screen time” paragraphs in bullets.	Discussion on home environment and goal sheets were good. Change “hand portion activity” to match the instructions.	“Screen time focused in all no just TV.” “My kids grasped it and they were able to really use it [hand measurement], you know, as far as even going out to eat, you know. They would say is this the right size for chicken? And we would say well look at your hand.” “It [home environment] gets lost in the workbook. Because this is actually really good because, you know, then I’m looking at it again. It’s really, really a good thing to have. But like the other worksheet that they hand out, 1 use that and 1 put that on the refrigerator to schedule things, you know.”	Changed “hand portion activity” to match the instructions and include race diverse pictures. Changed the Screen time section including all screen types no just TV and added a screen time calendar for families. Made the home environment quiz a “tear out.”
Chapter 4	Understand and making sense of a food label. Understand importance of games and how they can help them to improve health. Identify appropriate rewards for healthy behaviors and weight loss. Identify how they can be a role model for their family and friends.	Need to better outline ways to help to be successful.		“More information on the label part.” “1 think the food label message was really good. My son, he bought into that, you know, looking at calories and sugar and stuff like that, and my daughter as well (…) The one thing that 1 think we went over that 1 think we should have talked more about is the ingredients.” “It broke it down really good for her [my daughter], and she even now does it on her own.” “That’s something y’all should include is how much we need as far as our nutrients and sodium and how much a person should be taking in, because that really wasn’t in there.” “1 don’t know what 1 can do about sugar … 1 can’t just cut off all from my kids … 1 need an acceptable daily number …” “1 didn’t necessarily use the chart, but we learned from it; that making activity a reward is much better than ice cream, you know. And 1 learned, too, that my kids like it better. “More pictures”	Added more information on label section including sugar and sodium (the “5/10 rule”), and ingredients information. Added more pictures illustrating recommended actions.
Chapter 5	Reflect on eating choices in the last week and identify triggers to that lead to unhealthy eating. Identify movements and intensity routines of activities using music & dance that can help them to improve their family’s health. Identify strategies and resources to help their children to deal with bullying and teasing situations using the five- step strategy AWARE.	None		“1 think they were explained really well, especially this first page. It looks like a summary of everything, you know, with my plate and using their hand. The energy balance, you know, it was just a really nice summary before we moved on to the next section … 1 think that’s really important.” “1 would like to see more maybe pictures of some of the exercises. Some of the exercises that we did or maybe some that the program suggested that we do at home. That would be helpful.” “1 think the bullying part maybe, you know, just like the other part where you’re talking about elaborating on it a little more (…) Maybe a different picture or other pictures, because this triangle is good, but 1 think maybe we can do better, you know, to really drive home the whole bullying thing.” “1 like these /Choose [tracker] where you had the vegetables … well the fruit vegetables, grain, protein, dairy, and sometimes food … 1 think that’s great because that kind of its one thing to just think you’re doing all right. Versus you see it in print and you’re thinking, oh, 1 thought 1 had more vegetables than that. You know, it just brings it home. So 1 think this was a really good tool.”	Included an appendix which exercises that families did in the exercise class (circuit), and also which exercise that could be done at home Improved bulling section by reducing content and focusing in strategies. Included in all sections a “recap” paragraph at the beginning of each chapter.
Chapter 6	Describe relapse and recovery in a healthy eating and physical activity. Identify and practice exercises that can help to decrease stress. Identify strategies and resources to prevent relapse and to recovery after a relapse.	“1 feel this should be more goal based + strategies to avoid relapse” [X] suggested making title mention lapse/relapse more clear [Y] felt it was fine the way it was since the cycle graphic is just underneath. Not sure why the image about “desserts is stress backwards.” Seems counter to our main goals because the image with a cake did not support recommended behaviors and its purpose was unclear. One list exceeded seven bullets, but barely and the items in the list are short and easy to read.	Replace Desserts image with a culturally more appropriate image of health stress management methods such as those in the text next to the image.	“1 think you used very familiar words that you’re used to hearing about if you listen to anything about weight loss and stuff; they do talk about lapses and those kinds of things and healthy habits. You know, recovery is probably not a word I’ve heard used before with like lapses and relapses. That’s not a word 1 would necessarily think of.” “When we first got the book and 1 saw recovery, I’m like recovery? Is this talking about alcoholics?” “My take on it is that 1 like strong language like that because it makes me … it motivates me to get back on the wagon or whatever. Get me back on the trail to doing what 1 personally need to do, you know, to reach my goals. It means something positive to me because of my background. So 1 guess you guys, you have to find a balance between me and [participant Y] is it?” “1 think it was proactive [plan]. It’s always important to be proactive because, once something happens, you react and you don’t always have the best thought process. But if you have a plan in place, then you’re like, okay, 1 did this. Let me go to this and let me figure out where 1 should do from here to fix it. “1 like that [stress management] … And 1 like how you guys have this all set up, but 1 think maybe you could elaborate just a little bit more. Give us a little bit more tools of relaxation because, you know, us superwomen need all the help we can get, you know, to tackle the day.” “1 think this is helpful, you know, because it helps you to sustain, you know, all the lessons that you learned through the program. 1 have not actually cracked the book open and wrote down my strategies. 1 mean, 1 have it up in here and 1 have it in my heart. And 1 feel that I’m using the tools that you guys gave me. And this book is more of a reference for me, but 1 haven’t opened it up and started writing anything down.”	Replaced images with a culturally more appropriate image of health stress management methods. Review bullet list to contain less up to seven items. Reviewed content to be more goal based and focused in the family plan after the program. Kept lapse and relapse (strong language) to motivate the family planning for maintenance phase. Added more stress management tools for families. Included a family contract to stimulate parents keeping changes after program ends.

*Note.* POPS = Partnering for Obesity Planning and Sustainability; CAB = community advisory board; CCI = Clear Communication Index.
